# Supplementary material for: Raw material choices and technical practices as indices of cultural change: Characterizing obsidian consumption at ‘Mycenaean’ Quartier Nu, Malia (Crete)
Source: PLoS One. 2022 Aug 23;17(8):e0273093. doi: 10.1371/journal.pone.0273093 (PMC9397941; doi:10.1371/journal.pone.0273093)
Supplement: S1 Appendix — (PDF) [file pone.0273093.s002.pdf]

Carter and Kilikoglou - Raw Material Choices and Technical Practices as Indices of Cultural Change: Characterizing Obsidian Consumption at 'Mycenaean' *Quartier Nu*, Malia (Crete)

S2 Appendix: *Quartier Nu* database abbreviations used in S1 Table

<u>Blank</u>

B/F1 – blade-like flake with >80% cortical cover of dorsal surface

B/F2 – blade-like flake with 5-80% cortical cover of dorsal surface

B/F3 - blade-like flake with <5% cortical cover of dorsal surface

BL – blade

BL PD – blade from the full rhythm of production (*plein debitage* / prismatic)

BL REM COR – blade with remnant cortex on dorsal surface

BL REM CR – blade with remnant cresting scars on dorsal surface

CB – crested blade

CORE FRAG – core fragment

F1 – flake with >80% cortical cover of dorsal surface

F2 – flake with 5-80% cortical cover of dorsal surface

F3 - flake with <5% cortical cover of dorsal surface

PREP – core preparation flake

REJ – rejuvenation flake

REJ FC - rejuvenation flake from the core's face

REJ BACK - rejuvenation flake from the core's back

REJ BASE - rejuvenation flake from the core's base

REJ CT - rejuvenation flake from the core's platform (core tablet)
