# Supplementary material for: Raw material choices and technical practices as indices of cultural change: Characterizing obsidian consumption at ‘Mycenaean’ Quartier Nu, Malia (Crete)
Source: PLoS One. 2022 Aug 23;17(8):e0273093. doi: 10.1371/journal.pone.0273093 (PMC9397941; doi:10.1371/journal.pone.0273093)
Supplement: Attachment — Submitted filename: Response to reviewers.docx [file pone.0273093.s003.docx]

**Response to editor and reviewers: PONE-D-22-06920**

Raw Material Choices and Technical Practices as Indices of Cultural Change: Characterizing Obsidian Consumption At ‘Mycenaean’ Quartier Nu, Malia (Crete)

*Responses to editor*

- Paper has been reformatted according to PLOS One requirements. Table 3 is too wide however, even when the page is set to landscape, so hopefully your people can configure this appropriately.
- We have also corrected Table 3 by recalculating the missing values, fixing decimals according to precision and removing Nd, Ca and Ta which are not mentioned in the text anyway. We have also removed As from the elements determined (page 20, 2nd line) since it was not included in Table 3.
- Permission has been gained for reproducing the excavation plans, figure captions have been updated to include all pertinent information (those by Lopez, Le Bourdonnec, Milić, Labriola were fully paid for, i.e., the ownership now belongs to Carter).
- Laboratory protocols: the NAA facility is no longer functioning, so we see little point in uploading the protocols to Protocols.io
- Data availability – those data pertaining to the artifacts’ elemental profile, plus their context, and techno-typological characteristics are made fully available in this paper. Those data pertaining to the techno-typological characteristics, and context for the larger assemblage are not available to share publicly until the full excavation monograph has been published by the École Française d’Athènes.
- Reference list – removed those not quoted in text, and conversely added those mentioned in text, that were missing from the bibliography (as spotted by reviewer #1).
- Reference list – a couple of extra references relating to the *chaîne opératoire* approach were added to the bibliography (as requested by reviewer #1).
- Reference list – a couple of references were cut, as they were considered superfluous when rereading the text (e.g., Davaras 1971; Rehak and Younger 2001).

*Responses to Reviewer #1*

- Reference to Tixier et al., 1980 (and two other pertinent references to the concept of the *chaîne opératoire*) have been added as suggested.
- All bibliographic mistakes kindly listed by Reviewer #1 have been corrected, though four of them were referenced in Table captions: Driessen 1994 (Table 1); D’Annibale 2017; Karantzali 2016; Kardulias 1992 (Table 5).
- Their concern about the misuse of the term ‘functional’ is acknowledged, and any such references removed given the lack of a formal use-wear analysis.
- Fig. 4 – as requested, a scale and north arrow have been added. However, rather than add captions as to the location of kitchens etc. (detailing of multi-functional spaces has been the subject of project research, and is referenced: Driessen & Fiasse, 2011; Driessen et al., 2008), we have noted room numbers when mentioning specific features, such as the central court and freestanding kitchen, so that the reader can locate them.
- That Fig. 5 has previously been published is now acknowledged in the figure caption (“reproduced from Carter, 2004a; Fig. 21.6”). I have the rights to this image and its reproduction.
- Fig. 8 – concerns on lithic illustration conventions: (1) we now detail in the figure caption that all cross-sections were drawn at the artifact’s midpoint; (2) alas it is not possible to include the platform and profile (where missing) as the artifacts were part-destroyed during the analysis; (3) we respectfully note that the direction of blank knapping is indicated with reference to how the artifacts are oriented (this is standard), with the platform uppermost (following the Anglo tradition), the addition of extra symbols denoting flake direction we feel to be superfluous, and is not standard in our region of study (even amongst the classically trained French scholars, see the work of Catherine Perlès for example).
- The concern about the lack of available data – the main techno-typological study of the assemblage is being completed at present for the excavation monograph, in the same way that the sourcing and final studies of the *Quartier Mu* datasets were published dually. A statement to this effect has been added for clarification.
- By extent, while we understand the logic of the requested extra drawings of obsidian artifacts to detail the entire reduction sequence represented at *Quartier Nu* (cores etc.), we respectfully suggest that (a) at present the paper/data is neatly iterated in terms of retaining focus, (b) for us to show all the artifacts analyzed is in itself something of a rarity in sourcing studies, let alone a representative sample of the larger assemblage, and (c) such material will be detailed fully in the excavation monograph (again we point to the dual publication of the *Quartier Mu* datasets). We *do* however, now provide a histogram (Fig 6) that details the techno-typological classes of the larger Final Palatial obsidian assemblage.
- Inconsistencies regarding the size of the assemblage have been corrected; the statement about complete blades has been simplified to remove confusion.
- Repetitions – while structurally some repetition is felt to be necessary, we have cut some text to diminish this issue, in line with the reviewer’s concerns.
- Their statement concerning “it would have been interesting to enrich synchronic comparisons of material choices and production techniques with assemblages from 'normal' contemporary” – we agree, however, such a comparison really takes us away from our main foci, and to be honest there is precious little published in detail from Post Palatial sites away from the north coast for us to undertake such comparisons.

*Responses to Reviewer #2*

- All grammatical / stylistic errors kindly listed by Reviewer #2 have been corrected.
- The question as to whether chert sickle production use on the mainland related to there being greater amounts of that raw material available, is now addressed by the new text and references in the first half of the following line: “Given that chert is available naturally on Crete (e.g., Blitzer, 2004; Brandl, 2010), the obvious conclusion to draw from the rarity of such implements on Crete, is that people were using a different harvesting technology than their mainland contemporaries, with perhaps a greater reliance on bronze sickles to reap their cereals…”
- The question as to “When was Melos occupied, potentially with local production of cores to Crete and mainland Greece?” and whether we are only dealing with the movement of raw materials is addressed by a rewrite of that sentence to read: “In terms of typo-technological characteristics and the nature of the reduction sequence, the *Quartier Nu*, Mochlos and Chania assemblages are directly comparable, the communities importing at least some of their obsidian as raw nodules (potentially via their contemporaries at Phylakopi on Melos [Renfrew, 2007]) that they then shaped, reduced, and rejuvenated in the same manner.”
